# Influences of intergrowth structure construction on the structural and electrical properties of the BBT-BiT ceramics

**DOI:** 10.3389/fchem.2022.1089739

**Published:** 2022-12-13

**Authors:** Yuying Wang, Deyi Zheng, Runyu Mao, Xu Wang

**Affiliations:** College of Materials and Metallurgy, Gui Zhou University, Guiyang, China

**Keywords:** curie temperature, BBT-BiT, intergrowth structure, oxygen vacancy, electric properties

## Abstract

Bismuth Layer Structured Ferroelectrics (BLSFs) have always been an important research direction of high Curie temperature piezoelectrical ceramics, and the construction of intergrowth structure has been considered as an effective method to improve the electric properties of BLSFs. There are many literatures about intergrowth structure improving electrical performance, but few reports analyze the influence of the construction of intergrowth structure on the internal defects and electrical properties in BLSFs. In this study, (1-*x*) BaBi_4_Ti_4_O_15_ - *x* Bi_4_Ti_3_O_12_ ceramic samples with intergrowth bismuth layer structure were fabricated by a conventional solid-state reaction method, and the mechanism of the influence of intergrowth structure construction on the structure and electrical properties of BLSFs has been discussed. The crystal structure, phase composition, microstructure, dielectric and piezoelectric performance, relaxation behavior and AC conductivity of ceramic samples were systematically investigated. It has been found that the construction of intergrowth structure can significantly inhibit the generation of oxygen vacancies. The concentration of the oxygen vacancies plays an important role, and its reduction will lead to the inhibition of grain growth and the increase of the relaxation activation energy of ceramics. In addition, the intergrowth structure construction also affects the symmetry of ceramics in the *c*-axis direction, thus affecting the electrical properties of ceramics.

## 1 Introduction

The Aurivillius family of bismuth-layer structure ferroelectrics (BLSFs) as a promising candidate for high-temperature piezoelectric sensors is a development trend of piezoceramics in the future to overcome the high temperature working environment and enhance the applications in extreme environments (Wang, C. M et al., 2008; Zhang L. N et al., 2005). For example, Bi_4_Ti_3_O_12_ (referred to as BiT) is the first choice of piezoelectric material for high temperature piezoelectric vibration sensor (˃˃ 500°C) in the world at present, which is widely used in aerospace, nuclear energy and other fields to monitor the vibration of key equipment under high temperature and harsh environment (Zhang and Yu, 2011). The crystal structure of BLSFs is formed by pseudo-perovskite blocks (*A*_m-1_ *B*_m_O_3m+1_)^2-^ and bismuth layers (Bi_2_O_2_)^2+^ in regular alternate arrangement (Wong et al., 2017), where *A* is a mono-, di-, tri-valent (Na^+^, Ba^2+^, Sr^2+^, Ca^2+^, Bi^3+^, La^3+^, etc.) cations allowing 12-coordination, *B* is a six- coordination transition metal (Ti^4+^, Ta^5+^, Nb^5+^, Mo^6+^, W^6+^, Co^3+^, etc.) and *m* represents the number of octahedral layers (Hou et al., 2010; Li et al., 2019).

Bi_4_Ti_3_O_12_ (BiT, *m* = 3), the vital archetype of BLSFs, which has a higher Curie temperature (*T*_*c*_) of 675°C and strong ferroelectric polarization (Park et al., 1999). However, the BiT ceramics has a lower piezoelectric activity, the piezoelectric constant (*d*_33_) is less than 8 pC/N) (Hou et al., 2010; Xie et al., 2019). The BiT ceramic has an excellent piezoelectric constant (*d*_33_) of 20 pC/N and a large remnant polarization (2*P*_r_) above 18 μC/cm^2^ compared with other BLSFs ceramics after doping modification (Bokolia et al., 2015; Tang et al., 2018; Xie et al., 2019). BaBi_4_Ti_4_O_15_ (BBT, *m* = 4), a class of typical bismuth layer structure piezoceramics, showing a Curie temperature *T*_*c*_ value of 410°C, but the piezoelectric constant at a low level, about 7 pC/N (Qi et al., 2019). The piezoelectricity and ferroelectricity of BLSFs ceramics are undesirable, because easily evaporation of bismuth element at high temperature, forming oxygen vacancies to balance the charges (Jiang et al., 2020a). Thus, the applications of BLSFs are limited by their low piezoelectric activity, and the properties of BLSFs are usually improved by ions doping, construction of intergrowth structure, and preparation process adjustment.

The intergrowth BLSFs with relatively large values of remanent polarization (*P*_r_) are promising candidates to optimize the performance of BLSFs ceramics, which are composed of one-half the unit cell of an *m* member structure and one-half the unit cell of an *m* + 1 member structure (Noguchi et al., 2000; Yu et al., 2020). Kobayashi et al. (2004) studied that the *T*_c_ of the BBT-BiT (540°C) ceramics was between those of BiT (675°C) and BBT (410°C), and the value of spontaneous polarization (*P*_s_ = 52 μC/cm^2^) was larger than those of BiT and BBT ceramics. Ta^5+^ doped BBT-BiT intergrowth ceramics, the BaBi_8_(Ti_0.995_Ta_0.005_)_7_O_27_ showed an optimal piezoelectric performance with a *d*_33_ of 19 pC/N of a *T*_c_ about 475°C (Jiang et al., 2020b). And the introduction of (Li_0.5_Bi_0.5_)^2+^ ions into the BBT-BiT intergrowth ceramics increased the *d*_33_ from 8 to 18.5 pC/N and *T*_c_ from 480 to 633°C (Jiang et al., 2020a).

To improve the piezoelectric constant of BLSFs, the research on modification of BLSFs by synthesis techniques, ions doping and development of intergrowth structures have been highlighted by many researchers. There are many reports on the modification of BLSFs by constructing intergrowth structure, such as ions doped into intergrowth BLSFs (X. P. Jiang and Z. L. Jiang et al., 2020a; Jiang et al., 2020b) and component regulatory of intergrowth BLSFs (Kobayashi et al., 2004; B. Wu et al., 2018). However, few reports to analyze the micro mechanism of the influence of intergrowth structure construction on the internal defects of BLSFs and the reasons why these internal defects affect the electrical properties of BLSFs. In this study, the (1-*x*) BaBi_4_Ti_4_O_15_—*x* Bi_4_Ti_3_O_12_ (*x* = 0, 0.2, 0.4, 0.6, 0.8, 1) ceramics were prepared, our efforts were made to construct intergrowth structure and investigate the effect of intergrowth structure on microstructure and properties of (1-*x*) BBT—*x* BiT intergrowth ceramics with different content of Bi_4_Ti_3_O_12_. The results of XPS analysis and the calculated values of relaxation activation energy were used to analyze the effect of intergrowth structure on the oxygen vacancy concentration of ceramics. And the dielectric and piezoelectric properties study, AC impedance and conductivity analysis were used to study the micro mechanism of intergrowth structure improving electrical properties and investigated the influence rules of intergrowth structures on the microstructure and relaxation behavior of ceramics.

## 2 Experimental procedures

The (1-*x*) BaBi_4_Ti_4_O_15_ - *x* Bi_4_Ti_3_O_12_ (*x* = 0, 0.2, 0.4, 0.6, 0.8, 1) intergrowth ceramics were synthesized by a traditional solid-state reaction, which using metal oxides of BaCO_3_ (99%), Bi_2_O_3_ (99.999%) and TiO_2_ (98%). Bi_2_O_3_ exceeds 3 wt% to compensate for its evaporation at high temperature. All raw materials were ball-milled for 16 h and calcined at 810—850°C for 3 h, then sintered at 1020°C for 2 h after pressing the pre-sintered powders into *Ф* 12 × 1 mm pallets. The composition and crystal lattice of the samples were analyzed by X-ray diffraction (XRD, Model XPERT-PRO) with Cu Ka radiation. The microstructures were studied by Field emission scanning electron microscope (FE-SEM, Model JSM-5900). The grain size of all ceramics was calculated *via* the software of nano measure, and the bulk density (*ρ*_b_) of the ceramics was measured by the Archimedes method using a Solid Electronic Densimeter of Model MH-120C *via* the calculation of weight/volume. The transmission electron microscopy (TEM) was analyzed on a FEI Talos F200X system, while the X-ray photoelectron spectroscopy (XPS) was done using a Thermo ESCALAB 250XI spectrometer. Then, the piezoelectric constant (*d*_33_) was measured by a ZJ quasistatic piezo-*d*_33_ m (Model ZJ-3A) after poling in silicone oil at 50°C under a direct current (DC) electric field of 8–10 kV/mm for 15 min, and the dielectric loss (tan *δ*) was tested by an LCR tester (Model TH2618B). In addition, hysteresis loops (*P*–*E*) were measured at 10 Hz with a ferroelectric tester (Aixact TF Analyzer 2000), dielectric analysis at temperature (30—700°C) and impedance analysis at frequency (20 Hz–10 MHz) were also carried out *via* Wayne Kerr 6500B Impedance Analyzer (WK 6500B).

## 3 Results and discussion

### 3.1 Structural and morphological characterization

**FIGURE 1:**
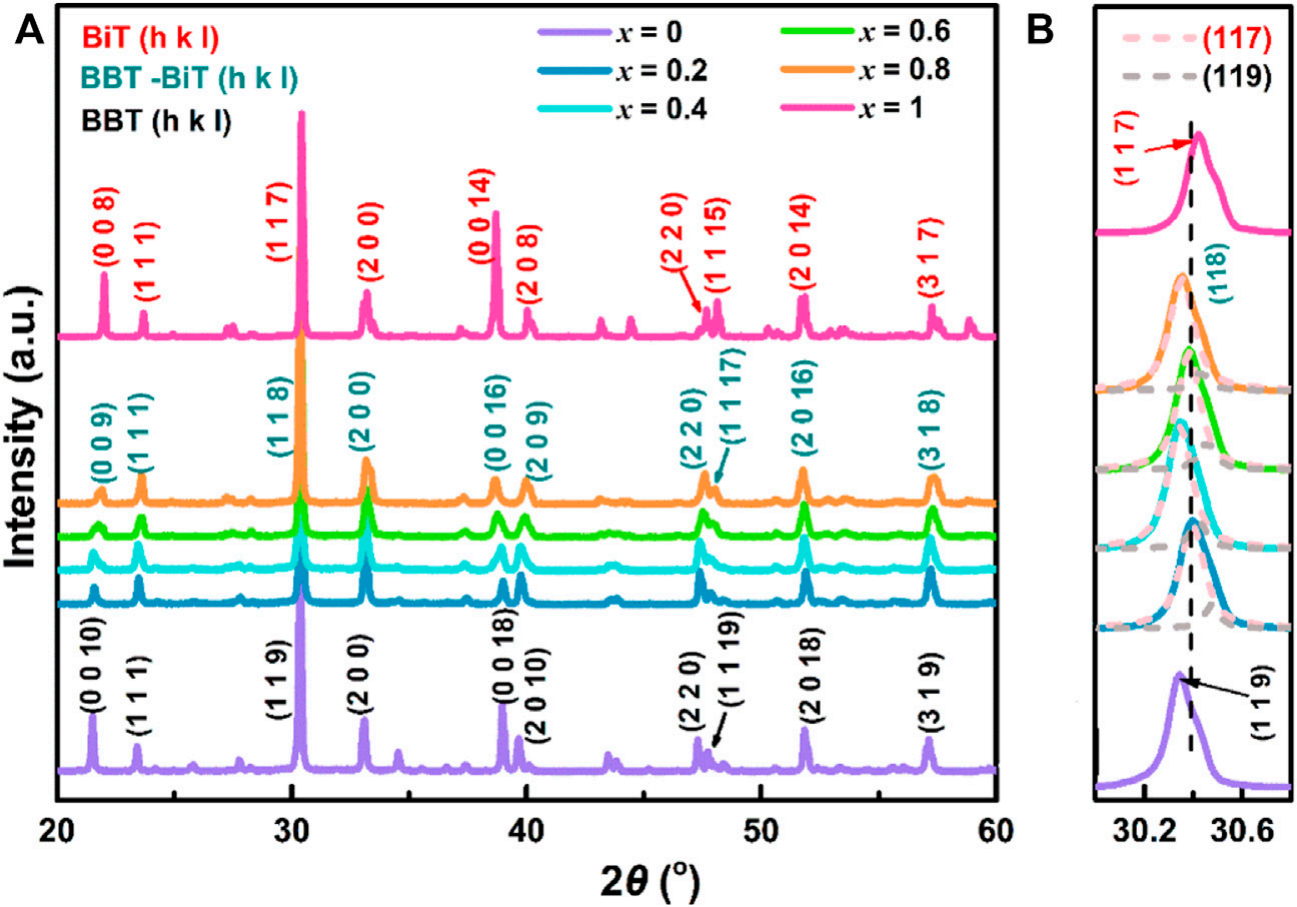
(Color online) **(A)** The room temperature XRD patterns showing the peak intensity relative to (1-*x*) BBT - *x* BiT ceramics in the 2θ range 20°–60°, **(B)** variation in the XRD peaks of BBT (119), BBT-BiT (118) and BiT (117).

The composition and crystal lattice of the (1-*x*) BaBi_4_Ti_4_O_15_—*x* Bi_4_Ti_3_O_12_ samples with a certain sintering temperature of 1020°C, the X-ray diffraction test are exhibited in Figure 1, *x* = 0–1. The results show that all diffraction peaks are in perfect agreement with the orthorhombic structure of BBT-BiT phase of intergrowth bismuth layered structure (BLSFs) with space group P2_1_am and no obvious second phase (Kobayashi et al., 2004; Wang W et al., 2008). Figure 1B shows the variation in the XRD peaks of BBT (119), BBT-BiT (118) and BiT (117) at 2*θ* = 30.2–30.6°, the dashed lines represent the peaks of Lorentzian profile function fitting. BBT with space group A2_1_am (*m* = 4, orthorhombic, JCPDS No. 35–0757) has the strongest peak (119), and BiT with space group B2cb (*m* = 3, monoclinic, JCPDS No. 72–1019) has the strongest peak (117). Although the position of X-ray diffraction peak shifts with the change of BiT content, the strongest peaks (118) of BBT-BiT (*m* ∼ 3.5, JCPDS No. 42–0053) are consistent with the crystal plane index (112*m* + 1) of the strongest peak in intergrowth BLSFs ceramics (Wang et al., 2010; Badapanda et al., 2019; Jiang et al., 2020a), which lie between the strongest peak (119) BBT and the strongest peak (117) of BiT. In addition, the peaks of BBT-BiT with the same Angle as that of BBT and BiT, but the peak intensity of BBT-BiT is lower than that of BBT and BiT. This indicates that the crystallinity of BBT-BiT is lower than that of BBT and BiT ceramics, and the conductivity of BBT- BiT ceramics is lower than that of BBT and BiT ceramics, which will be proved in the subsequent analysis.

**FIGURE 2:**
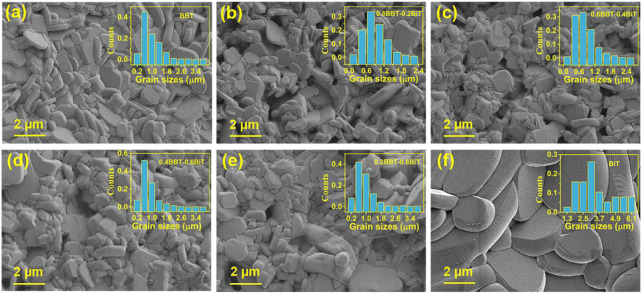
SEM images of (1-*x*) BBT—*x* BiT ceramics at a sintering temperature of 1020°C: **(A)** BBT, **(B)** 0.8BBT-0.2BiT, **(C)** 0.6BBT-0.4BiT, **(D)** 0.4BBT-0.6BiT, **(E)** 0.2BBT-0.8BiT, and **(F)** BiT.

Figure 2 shows the SEM images and grain sizes distribution on fracture surfaces of (1-*x*) BBT—*x* BiT ceramics with *x* = 0 (a), 0.2 (b), 0.4 (c), 0.6 (d), 0.8 (e) and 1 (f). Due to the high grain growth rate perpendicular to the *c*-axis during grain growth of bismuth layered compounds, all samples showed the typical plate-like grains typical random lamellar grain orientation and obvious anisotropy in grain structure (Suarez et al., 2001; Diao et al., 2013). The images also exhibit that most pores on the grain boundary rather than internal grain, which indicate that ceramics sintering is relatively dense. At the same time, the grain size of BiT ceramics is extremely larger than that of BBT ceramics, while BBT-BiT samples forming intergrowth structure that the two type of perovskite layers are interlaced and symbiotic along the *c*-axis (Luo et al., 2001). In addition, the SEM images also show that the BBT-BiT intergrowth ceramics have more holes and their grains growth are not sufficiently compared with those of the single BBT and single BiT ceramics.

**FIGURE 3:**
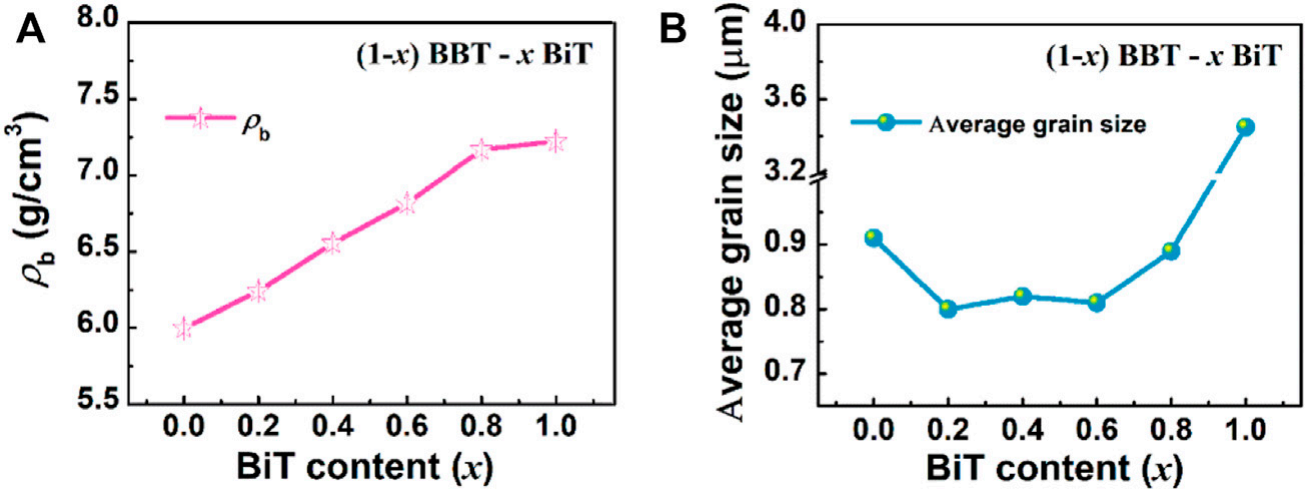
Evolutions in bulk density (*ρ*_b_) and average grain size as *x* value increase in (1-*x*) BBT - *x* BiT ceramics: **(A)** bulk density (*ρ*_b_), and **(B)** average grain size.

Figure 3A exhibits the evolutions in bulk density (*ρ*_b_) as *x* value increase in (1-*x*) BBT—*x* BiT ceramics. The bulk density (*ρ*_b_) of (1-*x*) BBT—*x* BiT ceramics increases with the *x* increasing, it may be caused by the differences in BBT and BiT ceramics systems. Figure 3B shows the average grain size of (1-*x*) BBT—*x* BiT ceramics. As mentioned in the XRD analysis that BBT-BiT ceramics have low crystallinity, which is usually shown as small grain size in SEM images. The average grain sizes of (1-*x*) BBT—*x* BiT (*x* = 0.2–0.8) ceramics are lower than those of BBT and BiT ceramics, combined with SEM images, these results indicate that the diffusion mechanism of BBT-BiT intergrowth ceramics play a role in the sintering process and inhibit the grain growth of the ceramic samples.

**FIGURE 4:**
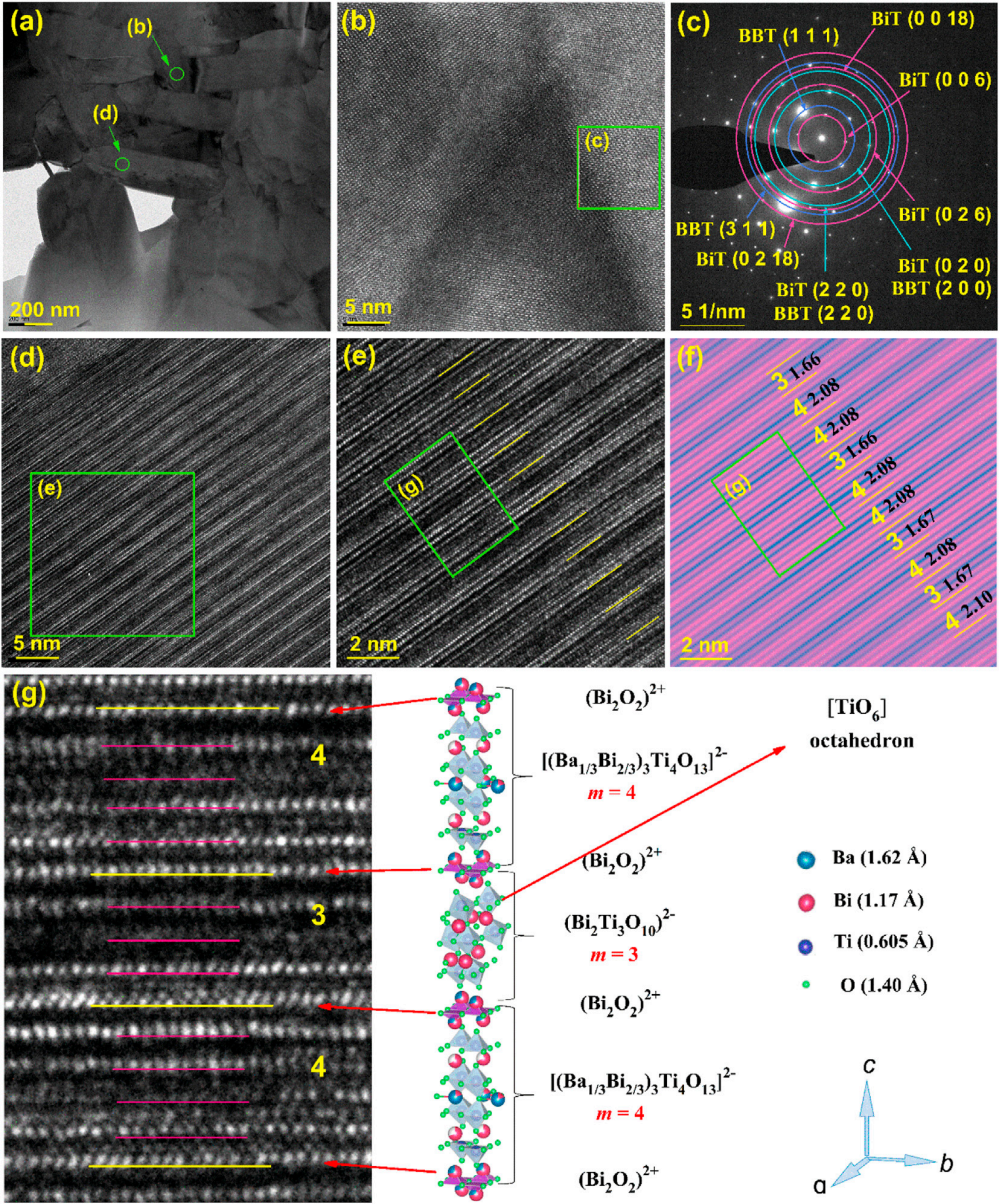
TEM images and schematic crystal structure of the 0.8BBT-0.2BiT ceramics: **(A)** Cross-sectional TEM image, **(B)** high-resolution Cross-sectional TEM image, **(C)** FFT image of Figure 4 **(B,D)** high-resolution Cross-sectional TEM image, **(E)** a partial larger version of Figure 4 **(D,F)** high contrast view with layer spacing of Figure 4 **(E,G)** a partial larger version of Figure 4 **(E)** with its schematic crystal structure.

Figure 4 shows the TEM images and schematic crystal structure of the 0.8BBT-0.2BiT ceramics. Figure 4A exhibits its cross-sectional TEM image, Figures 4B, D are the high-resolution cross-sectional TEM image. Figure 4C is the FFT image of Figure 4B, which shows the that both BBT and BiT phases coexist in the sample. Figures 4E, F are partial larger version of Figures 4D, F is a figure with increased contrast after color adjustment of Figure 4E, and Figure 4G is a partial larger version of Figure 4E. Figure 4F shows an irregular layered structure with alternating layers of three and four layers, and the layer spacing values are very close to half the length of the *c*-axis of three layers (3.3 nm) or four layers (4.2 nm). BBT ceramics are composed of bismuth oxygen layer (Bi_2_O_3_)^2+^ and four-layer pseudo-perovskite blocks [(Ba_1/3_Bi_2/3_)_3_Ti_4_O_13_]^2−^ regularly alternating, while BiT ceramics are composed of bismuth oxygen layer (Bi_2_O_3_)^2+^ and three-layer pseudo-perovskite blocks (Bi_2_Ti_3_O_10_)^2−^ regularly alternating. Combine Figures 4F, G, the schematic crystal structure of 0.8BBT-0.2BiT ceramics are formed by irregularly alternate arrangement of bismuth oxygen layer (Bi_2_O_3_)^2+^ and pseudo-perovskite blocks of three layers (Bi_2_Ti_3_O_10_)^2−^ or four layers [(Ba_1/3_Bi_2/3_)_3_Ti_4_O_13_]^2−^.

### 3.2 Dielectric and piezoelectric performance analysis

**FIGURE 5:**
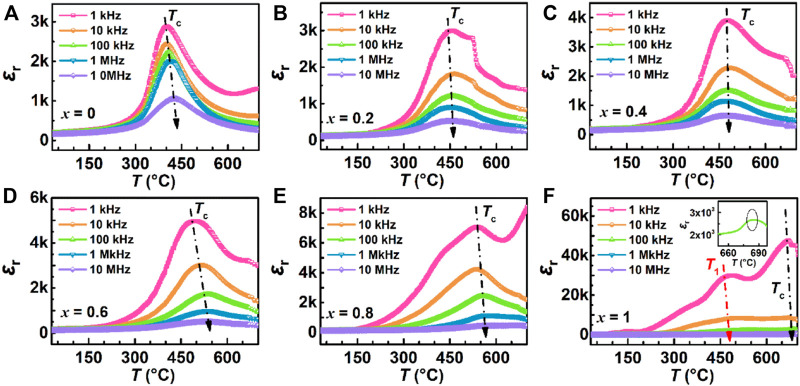
(Color online) Temperature dependence of dielectric constant for (1-*x*) BBT - *x* BiT ceramics at different frequencies (1 kHz–10 MHz): **(A)** BBT, **(B)** 0.8BBT-0.2BiT, **(C)** 0.6BBT-0.4BiT, **(D)** 0.4BBT-0.6BiT, **(E)** 0.2BBT-0.8BiT, and **(F)** BiT.

Figure 5 shows the temperature dependence of the dielectric constant *ε*_r_ for all samples from 30°C to 700°C at five frequencies as color online. It can be seen from *ε*_r_—*T* curves of all samples at these frequencies, the dielectric constants decrease with increasing frequency (that is phenomenon of frequency dispersion) and the peak of dielectric constants occur near Curie temperature *T*_c_. The *T*_1_ temperature of BiT ceramics in Figure 5F indicates that the sample has undergone phase transformation in the vicinity of this temperature. The flat dielectric constant in relation to temperature of relaxation ferroelectrics is due to the presence of polar nanoregions on the structure, which makes them in ergodic relaxor state (Ni et al., 2013). Meanwhile, the values of Curie temperature change at different frequencies and *T*_c_ increase with increasing frequency generally, except in Figures 5B, C. Due to the influence of space charge and conductivity, the dielectric constant tends to increase at low frequency and high temperature (Wang et al., 2022), so 100 kHz is selected as a frequency for subsequent experimental analysis.

**FIGURE 6:**
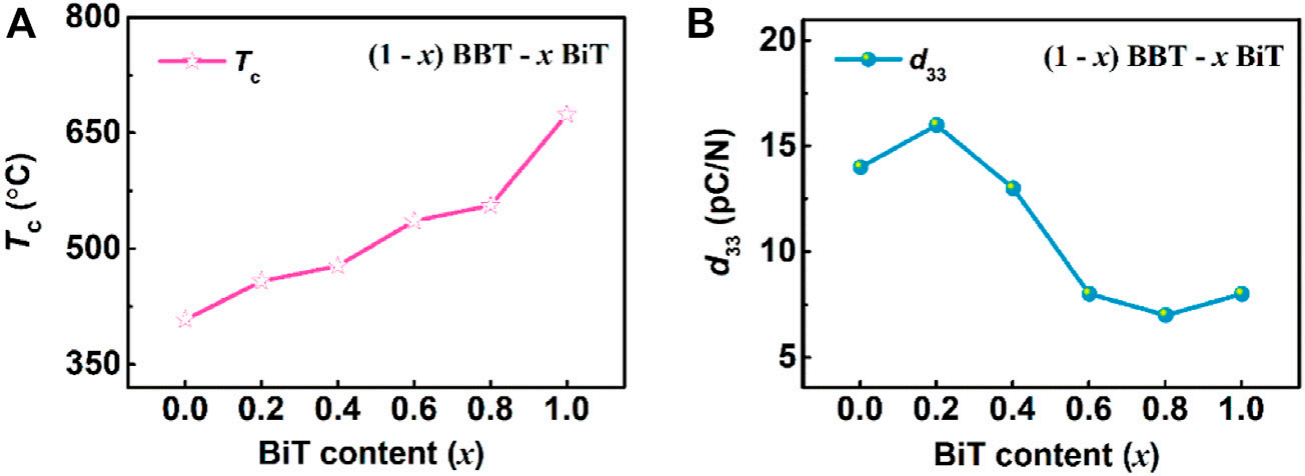
(Color online) The variation of Curie temperature *T*_c_, piezoelectric constant *d*_33_, coercive field *E*_c_ and remnant polarization *P*_r_ for (1-*x*) BBT—*x* BiT ceramics with different BiT content (*x*): **(A)** *T*_c_, **(B)** *d*_33_.

Figure 6 exhibits the curves of Curie temperature *T*_c_ and piezoelectric constant *d*_33_ of (1-*x*) BBT—*x* BiT ceramics with different BiT content (*x*), respectively. As the *T*_c_—*x* curve at 100 kHz shown in Figure 6A, the Curie temperatures *T*_c_ (458–556°C) of BBT-BiT intergrowth ceramics are between BBT (408°C) and BiT (674°C), which increase with the increase of BiT content *x*. Lattice distortion is the main influencing factor of Curie temperature (*T*_c_) of bismuth layered ceramics, which can be expressed by tolerance factor (*t*), as shown in law (Eq. 1) (Suarez et al., 2001).

$$t=\frac{\left(R_{A}+R_{O}\right)}{\sqrt{2}\left(R_{A}+R_{O}\right)}$$ (1)

where, *R*_A_, *R*_A_ and *R*_O_ are the ionic radii of A-site, B-site and O^2-^ respectively. The smaller the tolerance factor *t*, the higher the Curie temperature *T*_c_ of the ceramics. At this work, the general formula is (Bi_2_O_2_)^2+^(A_m-1_B_m_O_3m+1_)^2−^ for bismuth-layered structure ferroelectrics (BLSFs), the B-site is occupied by Ti^4+^ with an ionic radius of 0.605 Å, while the A-site is Bi^3+^ with an ionic radius of 1.17Å for Bi_4_Ti_3_O_12_ (BiT) ceramics and A-site is (Ba2+1/3Bi3+2/3) for BaBi_4_Ti_4_O_15_ (BBT) ceramics with an ionic radius of 1.32 Å. Thus, the radius of A site ion for (1-*x*) BaBi_4_Ti_4_O_15_ - *x* Bi_4_Ti_3_O_12_ intergrowth ceramics decreases with the increase of BiT content (*x*). With the increase of *x*, the increase of radius of A-site leads to decrease *t*, so *T*_c_ gradually increases, indicating that the BBT-BiT intergrowth structure of BLSFs increase Curie temperature compared with single BBT ceramics.

As shown in Figure 6B, the piezoelectric constants *d*_33_ increase first and then decrease with the increase of *x* of BBT-BiT intergrowth ceramics, show an opposite trend in changes, compared with that of *T*_c_. The intergrowth structure of BLSFs with *m*/*m*+1 layers are composed of *m* layers and *m*+1 layers, two-phase lattice distortion occurs due to the mismatch of lattice parameters, resulting in the formation of new intergrowth structural phases to match the lattice (Yang et al., 2018). The intergrowth structure, which affects the symmetry of ceramic *c*-axis direction and the properties of material. And the intergrowth structure construction also effects oxygen vacancy concentration, which will affect the deflection of ferroelectric domains, thus affecting the piezoelectric properties of BBT-BiT ceramics. At the same time, the maximum value of *d*_33_ for BBT-BiT ceramics is obtained at *x* = 0.2, the piezoelectric constant *d*_33_ is 16 pC/N and its corresponding Curie temperature *T*_c_ is 458°C. Combined with the SEM results, it can be found that the BBT-BiT intergrowth ceramic (especially 0.8BBT-0.2BiT sample) are with smaller grain size and more pores than the BBT and BiT samples. Therefore, the oxygen vacancy concentration can be controlled through many methods, such as ions doping and vacuum sintering, to further improve the grain growth and electrical properties.

### 3.3 XPS spectra study

**FIGURE 7:**
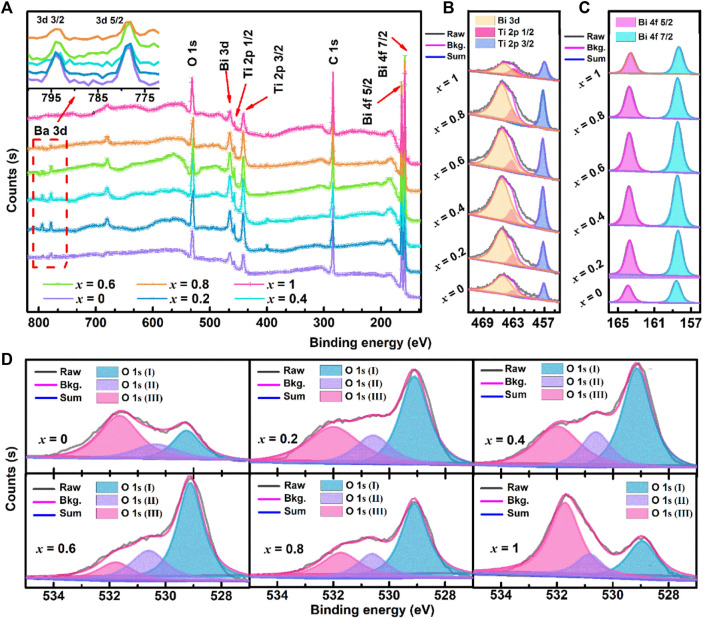
(Color online, Bkg. is short for Background) XPS spectra for (1-*x*) BBT—*x* BiT ceramics with different BiT content (*x*): **(A)**, XPS Survey, **(B)** O 1s scan, **(C)** Bi 4f scan, and **(D)** Bi 3 days and Ti 2p scan.

Figure 7 shows the XPS survey spectra and O 1s scan of (1-*x*) BBT—*x* BiT ceramics. As shown in Figure 7A, the resolution spectrum of Bi 3 days has 3 days 3/2 and 3 days 5/2 doublets in the binding energy range of 800–770 eV, and the change of peak area reflects the decrease of Ba 3 days content with the increase of BiT content (*x*). Bi 4f, Bi3d and Ti 2p spectra increase first and then decrease in the peak areas as shown in Figures 7B, C. For (1-*x*) BBT - *x* BiT intergrowth ceramics (*x* = 0.2–0.8), the binding energy of Bi 4f, Bi3d and Ti 2p shift not significantly with the *x* increasing, compared with those of BBT and BiT ceramics, which also shows that the intergrowth bismuth layered ceramics did not generate other impurity phases.

To further confirm the sources of oxygen vacancy in the samples, the peaks of O 1s scan are fitted into three peaks as shown in Figure 7D. Take BBT ceramics for example, the Lattice oxygen ions (O_Lattice_) are represented as O 1s (I) peak (529.29 eV), the None-lattice oxygen ions (O_Non-lattice_) are represented as O 1s (II) peak (530.38 eV) induced by oxygen deficiency from deviation of BaBi_4_Ti_4_O_15_ stoichiometric ratio in oxygen defect region and the O 1s (III) peak (531.68 eV) generated by the organic oxygen from the circumstance of the samples’ surface (Yoo et al., 2012; Jaim et al., 2017; Reddy et al., 2019). Table 1 shows the values of the integral Area and Area percentage content of oxygen peaks from the above different sources, and the ratio of O_Non-lattice_/O_Lattice_.

**TABLE 1:** Bind Energy, Peak Area and its Area percentage content of oxygen peaks, and ratio of O_Non-lattice_/O_Lattice_ for all samples.

BBT-*x*BiT		*x* = 0	*x* = 0.2	*x* = 0.4	*x* = 0.6	*x* = 0.8	*x* = 1
O^2-^	B. E. (eV)	529.29	529.08	529.15	529.12	529.10	528.95
	Area	23598.27	69507.45	80410.92	77039.91	61366.95	35073.94
	Area %	26.66	46.78	50.46	58.89	53.23	28.68
O^2^/O^−^	B. E. (eV)	530.38	530.49	530.56	530.60	530.61	530.83
	Area	17173.21	29346.75	29817.38	33770.72	22651.98	19011.79
	Area %	19.40	19.75	18.71	25.82	19.65	15.54
O1s	B. E. (eV)	531.68	531.98	531.95	531.78	531.73	531.71
	Area	47730.11	49721.93	49113.4	20003.07	31261.56	68223.16
	Area %	53.93	33.47	30.82	15.29	27.12	55.78
O_Non-lattice_/O_Lattice_		2.75	1.14	0.98	0.70	0.88	2.49

Bismuth volatilizes easily at a high sintering temperature of 1000–1100°C for BLSFs, resulting in forming Bi vacancies ( $V_{BI}^{\prime \prime \prime }$ ) and oxygen vacancies ( $V_{O}^{••}$ ) that can be expressed by Kröger–Vink formula (2) (Qiao et al., 2016):

$$2Bi_{Bi}^{\times }+3O_{O}^{\times }\Leftrightarrow Bi_{2}O_{3}\left(gas\right)+2V_{BI}^{\prime \prime \prime }+3V_{O}^{••}$$ (2)

where $Bi_{Bi}^{\times }\;and\;O_{O}^{\times }$ present electrically neutral vacancy of Bi and O, respectively. According to the O_Non-lattice_/O_Lattice_ values of the (1 − *x*) BBT − *x* BiT ceramics in Table 1, there are more oxygen vacancy defects in single BBT (2.75) and BiT (2.49) BLSFs, indicating that the intergrowth structure of BLSFs can inhibit the generation of oxygen vacancies. Oxygen vacancies promote ions to diffusion and transfer during sintering, improving the sintering speed of ceramics to promote the growth of grains (Tewari et al., 2014; Tang et al., 2020), which are also consistent with the SEM images (Figure 2) and average grain size (Figure 3B) results.

### 3.4 Impedance analysis

**FIGURE 8:**
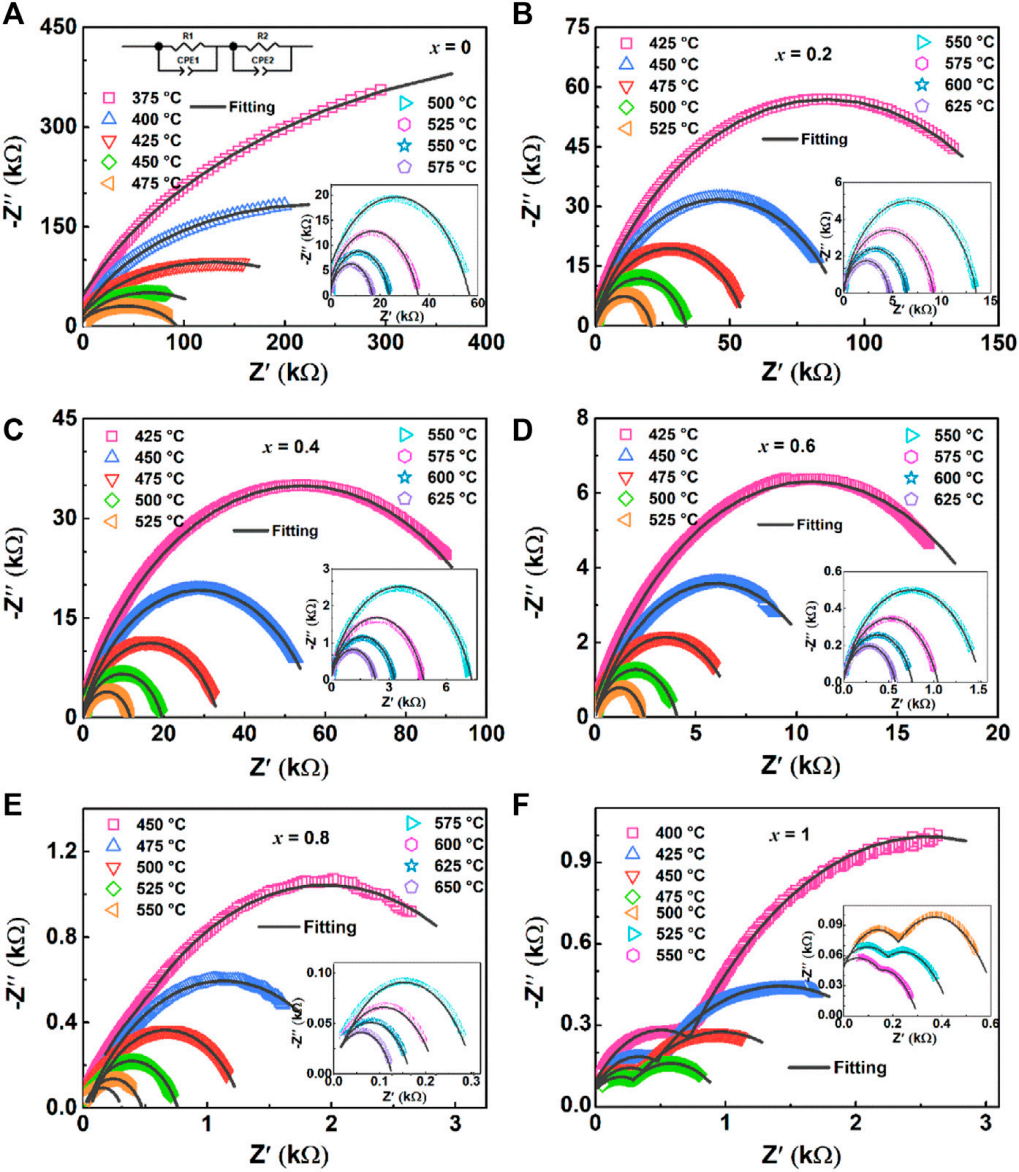
(Color online) Complex impedance spectra for (1-*x*) BBT—*x* BiT ceramics at various temperatures: **(A)** BBT, **(B)** 0.8BBT-0.2BiT, **(C)** 0.6BBT-0.4BiT, **(D)** 0.4BBT-0.6BiT, **(E)** 0.2BBT-0.8BiT, and **(F)** BiT.

Figure 8 shows Z′-Z″ complex impedance spectra collected under various temperatures with the frequency of 100 Hz–10 MHz, the impedance spectra selected the temperature range of the impedance circle that can be obtained by the sample. These Z′-Z″ spectra are fitted with the equivalent circuit as shown in Figure 8A, which can be composed of approximately two superimposed semicircles, reflecting the impedance contribution of the grain (high-frequencies arc) and grain boundary (low-frequencies arc) to the materials (Dhak et al., 2008). Figures 8A–E show that the Z′-Z″ spectra of ceramics are approximately single semicircles, which mainly represent the dominant role of grain boundary characteristics, and the interaction between grain, grain boundary and ceramic electrode interface (Lin and Chen, 2009). Moreover, the BiT ceramic exhibits grain and grain boundary characteristics as shown in Figure 8F.

For all samples, the radius of curvature decreases with increasing temperature for the Z′-Z″ complex impedance spectra, indicating that the resistivity of the sample has a negative temperature coefficient (Fujii et al., 2002). Meanwhile, Z′ decreases gradually with the increase of BiT content at the same temperature. Asymmetric in all characteristic wide peaks at measured temperatures for all samples indicates non-Debye type relaxation processes (Pradhani et al., 2018), and the values of resistance, capacitance and relaxation time for the bulk and grain boundary was listed in Table 2.

**TABLE 2:** Fitting results of all ceramics at the temperatures of 450°C, 500°C and 550°C: Resistance (*R*_g_), capacitance (*C*_g_), relaxation time determined (*τ*_g_) and relaxation activation energy (*E*_g_) for bulk (grain); resistance (*R*_gb_), capacitance (*C*_gb_), relaxation time determined (*τ*_gb_) and relaxation activation energy (*E*_gb_) for grain boundary.

(1-*x*) BBT—*x* BiT		*R*_g_ (kΩ)	*C*_g_ (nF)	*τ*_g_ (10^–5^ s)	*E*_g_	*R*_gb_ (kΩ)	*C*_gb_ (nF)	*τ*_gb_ (10^–5^ s)	*E*_gb_
*x* = 0	450°C	73.37	1.37	10.0420	—	130.08	1.94	25.2244	1.50
	500°C	21.75	0.70	1.5199	—	54.38	1.19	6.4837	1.50
	550°C	14.20	0.47	0.6635	—	22.89	0.66	1.5199	1.50
*x* = 0.2	450°C	60.20	1.83	11.0108	—	91.01	2.59	23.5407	1.32
	500°C	19.06	1.26	2.4089	—	35.18	1.89	6.6347	1.46
	550°C	0.93	0.90	0.8746	—	12.94	1.12	1.4515	1.46
*x* = 0.4	450°C	22.62	1.53	3.46	—	55.80	2.85	15.9155	1.30
	500°C	9.55	1.29	1.24	—	19.81	21.16	4.2837	1.59
	550°C	4.09	0.87	0.36	—	7.41	1.36	1.0042	1.59
*x* = 0.6	450°C	6.67	1.31	0.8746	—	12.38	2.51	3.1033	1.21
	500°C	2.80	1.46	0.4091	—	4.09	2.14	0.8746	1.21
	550°C	1.43	1.34	0.1913	—	1.57	1.50	0.2354	1.45
*x* = 0.8	450°C	2.69	1.63	0.4383	—	4.03	2.49	1.0042	1.32
	500°C	1.37	2.31	0.3176	—	1.27	1.99	0.2522	1.32
	550°C	0.56	2.28	0.1264	—	0.47	1.69	0.0798	1.32
*x* = 1	450°C	0.54	0.36	0.0196	0.43	1.92	20.79	3.9978	0.89
	500°C	0.30	0.31	0.0094	1.69	0.74	61.99	0.4590	0.89
	550°C	0.11	0.18	0.0020	1.69	0.34	16.34	0.0552	2.36

**FIGURE 9:**
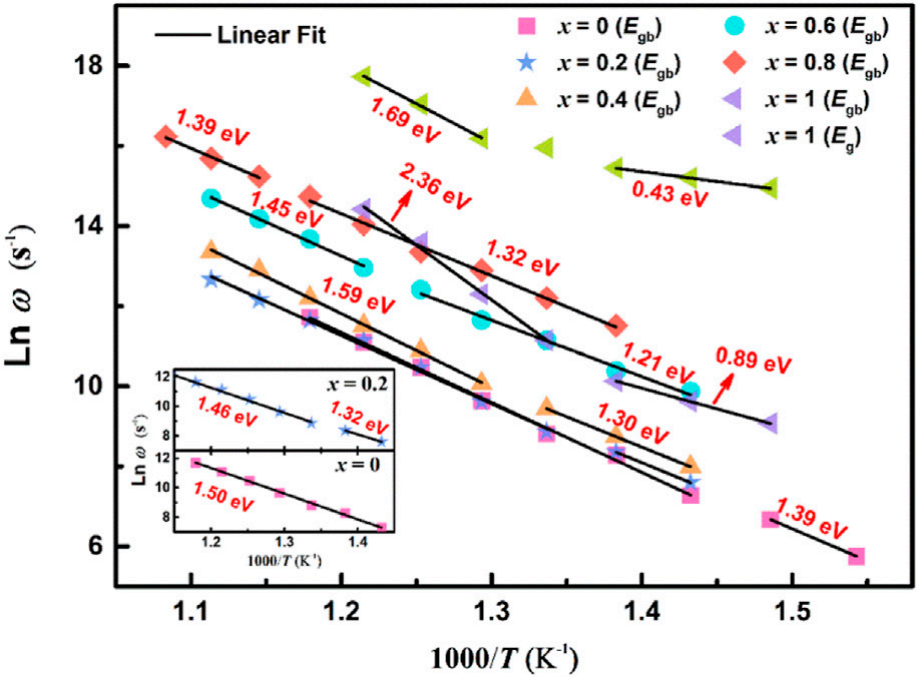
(Color online) The relaxation frequency Arrhenius diagram for (1-*x*) BBT - *x* BiT ceramics at different temperatures.

To confirm the relaxation mechanisms of the ceramics, relaxation frequencies (*ω*_max_ (*T*)) in relation to temperature ln *ω* - 1/*T* are plotted in Figure 9. The relaxation activation energy (*E*_relax_) is calculated by Arrhenius equation according to the slopes obtained from the linear fitting of the plots, this law (Eq. 3) as follow (Qiao et al., 2016):

$$\omega_{\max}\left(T\right)=\omega_{0}\exp\left(-\frac{E_{\mathrm{relax}}}{k_{B}T}\right)$$ (3)

where *T* is the temperature in Kelvin scale, *ω*_0_ is the pre-exponential factor, and *k*_B_ is the Boltzmann constant.

Figure 9 shows ln *ω* - 1/*T* relaxation frequency Arrhenius diagram of all ceramics at different temperatures, and the values of activation energy *E*_relax_ (*E*_g_ and *E*_gb_ are relaxation activation energy of grain and grain boundary, respectively) of the relaxation process are summarized in this figure and Table 2. The relaxation activation energy values of grains of (1-*x*) BBT - *x* BiT (*x* = 0–0.8) ceramics are no longer given here, because of the conduction mechanisms of these samples are mainly from the grain boundary contribution. In the thermal excited state, the oxygen vacancy will undergo single and double ionization and generate free electrons, as shown in Eq. 4 and Eq. 5 (Wu and Wang, 2010):

$$V_{O}\Leftrightarrow V_{O}^{•}+e^{\prime }$$ (4)

$$V_{O}^{•}\Leftrightarrow V_{O}^{••}+e^{\prime }$$ (5)

Where $V_{O}$ is unionized oxygen vacancies, $e^{\prime }$ is free electron, and $V_{O}^{\;•}\;and\;V_{O}^{••}$ present singly and doubly ionization oxygen vacancy, respectively. In addition, the activation energy of the double ionized oxygen vacancy is approximately 0.6–1.2 eV, while that of the single ionized oxygen vacancy is less than 0.3–0.5 eV (Ma et al., 2021).

The BBT sample above the Curie temperature *T*_c_ (408°C) with an activation energy *E*_gb_ of 1.50 eV between 425 and 575°C, and the BBT sample below *T*_c_ with an activation energy *E*_gb_ of 1.39 eV between 375 and 400°C. This figure exhibits different conduction modes before and after ferroelectric phase transition for the (1-*x*) BBT - *x* BiT (*x* = 0.2–0.8) intergrowth ceramics. The activation energy values of these ceramics are lower at low temperatures and low frequencies before the ferroelectric phase transition, while the values are higher of these ceramics at high temperatures and high frequencies after the ferroelectric phase transition. These values of *E*_relax_ are much larger than that of oxygen vacancy activation energy (more than 1.0 eV), indicating that the movement of oxygen vacancy is no longer the main conduction mode at high temperature and the reduction of oxygen vacancy content (Steinsvik et al., 1997).

The BiT ceramics undergo phase transformation near temperature *T*_1_ (Figure 5F) to exhibit various values of *E*_relax_. At the temperature of 400–450°C, it amounts to 0.43 eV at lower frequency and 0.89 eV at higher frequency, illustrating that the conductivity mainly comes from the conductivity of single ionized oxygen vacancy at lower frequency, and the carrier migration at higher frequency is mainly manifested by oxygen vacancy overcoming potential barrier transition from high energy level to low energy level (Chan et al., 1982; Raymond et al., 2005). At higher temperature of 475–550°C, BiT ceramics show great values of relaxation activation energy, and the dominant modes of conduction are cationic vacancies rather than oxygen vacancy at higher temperature (Guiffard et al., 2005).

Compared with BiT ceramics, the intergrowth structure of BBT-BiT ceramics improves the activation energy of BLSFs and reduces the oxygen vacancy concentration of BLSFs. The more oxygen vacancies can increase the leakage current of the ceramics, resulting in the incomplete polarization of the ceramic and the decrease of the piezoelectric constant, which in keeping with the electric properties results (Figure 6).

Table 2 exhibits the fitting values of resistance (*R*_g_ and *R*_gb_), capacitance (*C*_g_ and *C*_gb_), relaxation time determined (*τ*_g_ and *τ*_gb_) and relaxation activation energy (*E*_g_ and *E*_gb_) for bulk and grain boundary at various temperatures of 450°C, 500°C and 550°C, respectively. The resistance and relaxation activation energy values of the grain boundary are greater than those of the grain (*R*_g_ > *R*_gb_, *E*_g_ > *E*_gb_), and the resistance and relaxation time of ceramics gradually decrease with the increasing of BiT content *x* and test temperature.

**FIGURE 10:**
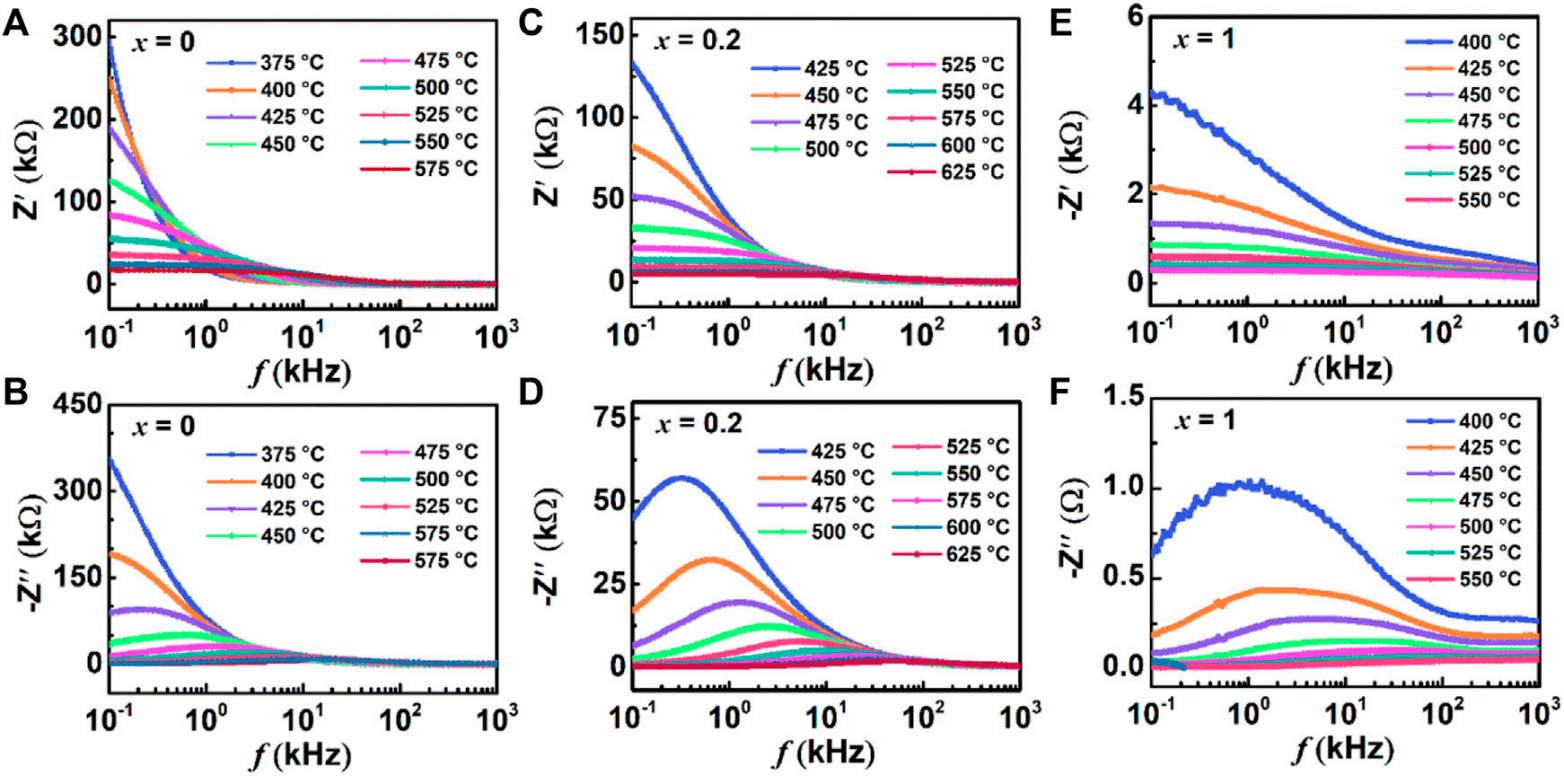
(Color online) Frequency variation of Zʹ and Zʺ from 100 Hz ~ 1 MHz at various temperatures for(1-*x*) BBT - *x* BiT ceramics: **(A,B)** BBT **(C,D)** 0.8BBT-0.2BiT, and **(E,F)** BiT.

The real part of impedance responses for BBT (a), 0.8BBT-0.2BiT (c), and BiT (e) BiT, as well as imaginary part of impedance responses for BBT (b), 0.8BBT-0.2BiT (d), and BiT (f) BiT are presented in Figure 10. The amplitude of Zʹ decreases with increasing temperature, indicating an increase of the AC conductivity of ceramics with increasing temperature, as shown in Figures 10A, C, E (Rafiq et al., 2015). With the temperature increasing, the maximum values of Zʺ are shifted and broadened at a higher frequency, which indicates the presence of relaxation processes with temperature-dependent, as shown in Figures 10B, D, F (Macdonald and Jhonson, 2005). There are two relaxation peaks at the frequency range of BiT ceramics as shown in Figure 10F, the lower and higher frequencies regions correspond to the impedance response of the grain-boundary and grain respectively.

### 3.5 AC conductivity analysis

To study further on the conduction mechanism of ceramics, the AC conductivity (*σ*_ac_) (Majhi et al., 2009) and conductance activation energy (*E*_con_) (Gupta et al., 2018) of samples conform to the following Eq. 6 and Eq. 7, respectively.

$$\sigma_{\mathrm{ac}}=\left(\frac{Z^{\prime }}{{Z^{\prime }}^{2}+Z{\prime \prime }^{2}}\right)\left(\frac{d}{A}\right)$$ (6)

**FIGURE 11:**
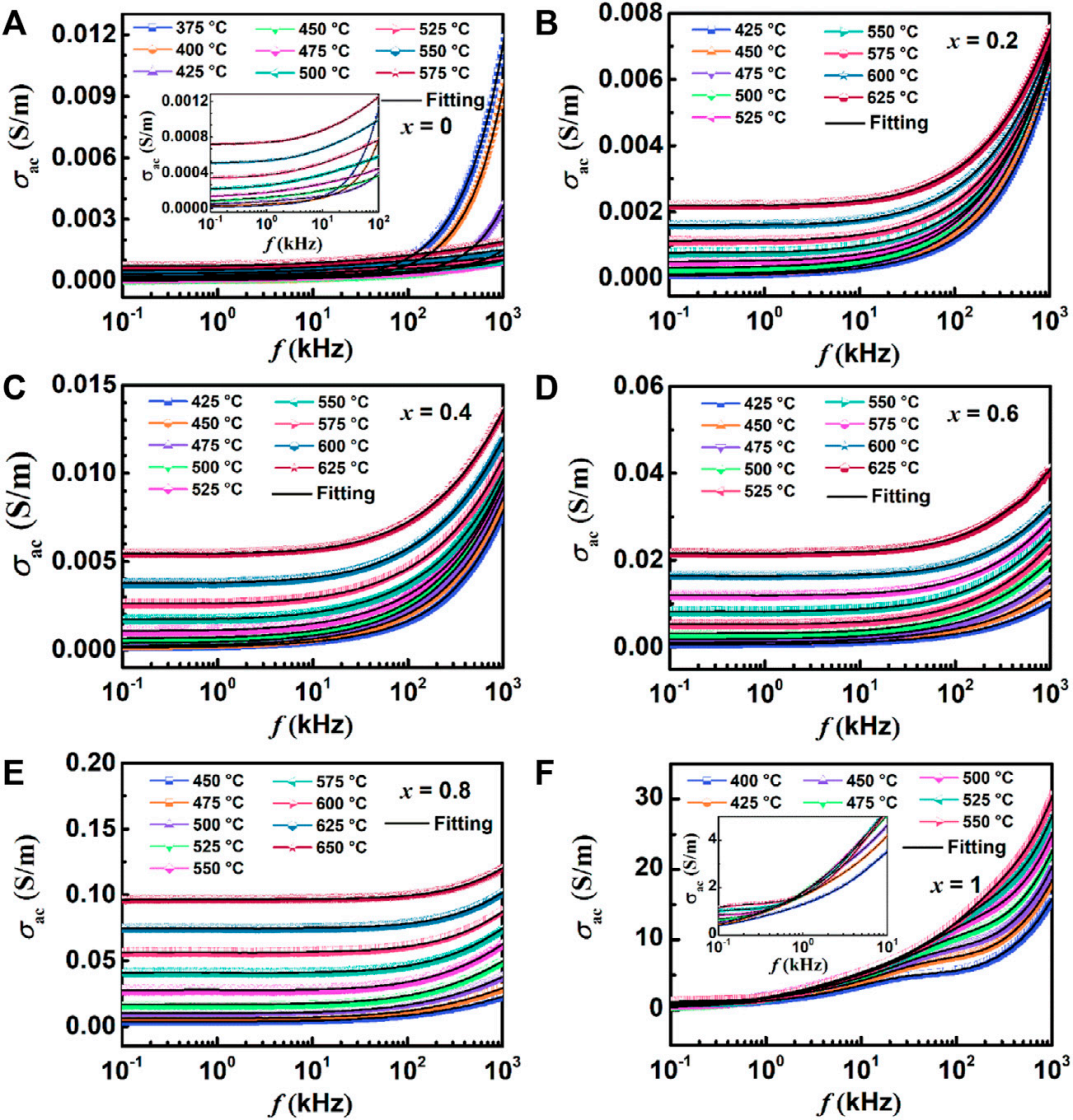
Variation of AC conductivity and frequency at different temperatures: **(A)** BBT, **(B)** 0.8BBT-0.2BiT, **(C)** 0.6BBT-0.4BiT, **(D)** 0.4BBT-0.6BiT, **(E)** 0.2BBT-0.8BiT, and **(F)** BiT.

Where *d* and *A* are the diameter and area of the sample, respectively. And the AC conductivity *σ*_ac_ diagrams of all samples are shown in Figure 11.

$$\sigma_{\mathrm{ac}}=\sigma_{0}\exp\left(-\frac{E_{\mathrm{con}}}{k_{B}T}\right)$$ (7)

**FIGURE 12:**
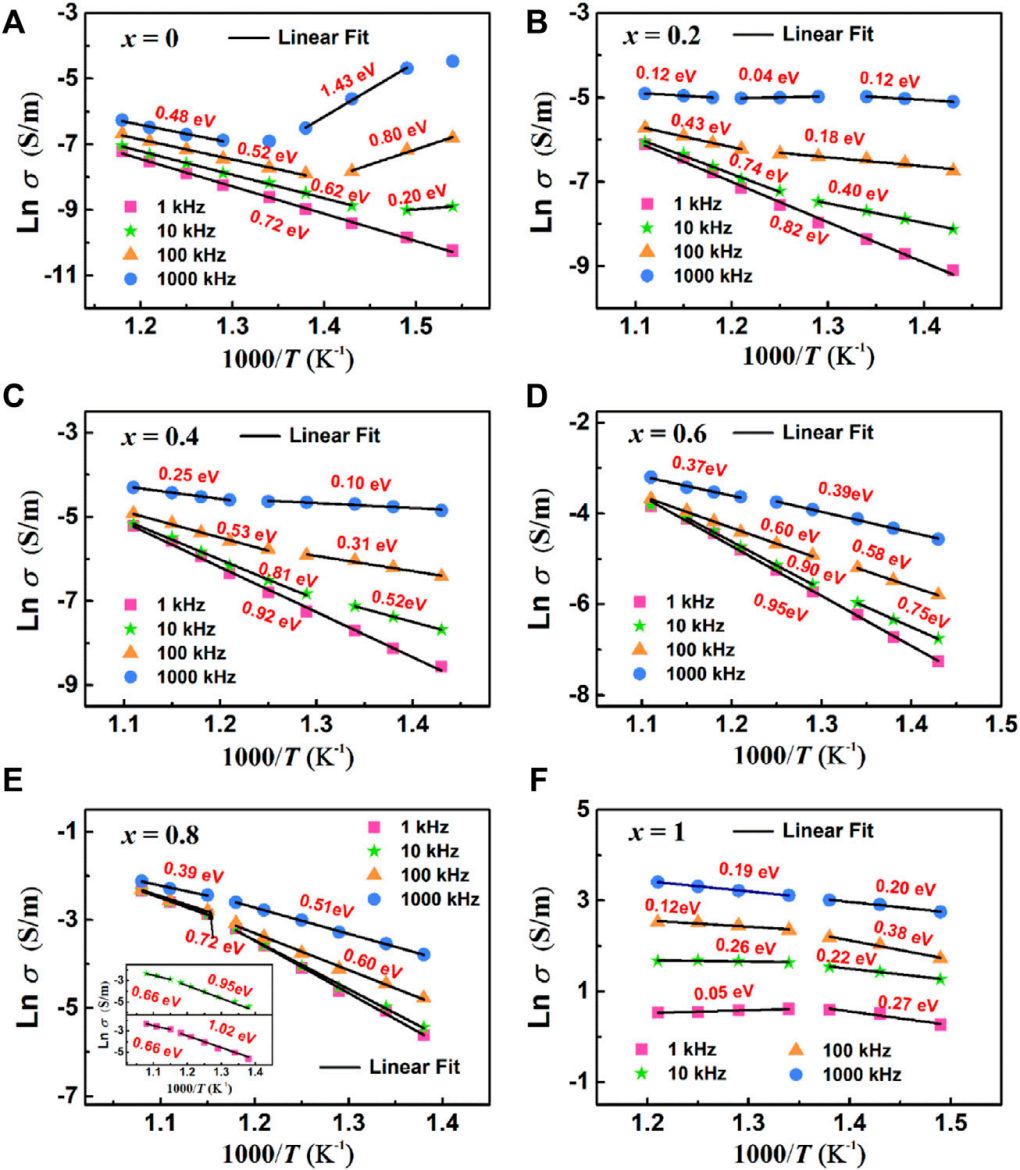
Arrhenius plots of AC conductivity at different frequency: **(A)** BBT, **(B)** 0.8BBT-0.2BiT, **(C)** 0.6BBT-0.4BiT, **(D)** 0.4BBT-0.6BiT, **(E)** 0.2BBT-0.8BiT, and **(F)** BiT.

Where *σ*_0_ is the pre-exponential term. The values of conductance activation *E*_con_ for all samples are summarized in Figure 12.

Figure 11 shows the variation of AC conductivity and frequency of all ceramics at different temperatures. The AC conductivity of the (1-*x*) BBT - *x* BiT ceramics increases with *x* increasing, because the conductivity of BiT ceramics itself is higher than that of BBT ceramics. The values of conductivity for 0.2BBT-0.8BiT ceramics with the optimal performance are in the range of 5.6 × 10^–5^–6.8 × 10^–3^ S/m below the curie temperature of 475°C (at frequencies of 10^2^–10^6^ Hz), which is lower than that of single BiT ceramics (0.85–22.74 S/m). Combined with the above analysis results, it can be concluded that the decrease of oxygen vacancy concentration has the main contribution to the decrease of ceramic conductivity. Meanwhile, the AC conductivity increases with the increase of temperature, indicating that conductance is related to thermally activated carriers (mainly oxygen vacancies and local carriers). Moreover, the conductivity of the ceramic is basically unchanged at low frequency, and the conductivity increases with increasing temperature and frequency at higher frequencies by the formation of AC conductance by the charge hopping process (Raymond et al., 2005).

Figure 12 shows the Arrhenius plots of AC conductivity of (1-*x*) BBT—*x* BiT ceramics (*x* = 0–1) at different frequencies, and the activation energy values of ceramics were calculated from the Ln *σ*—*T* linear fitting data. As for the (1-*x*) BBT—*x* BiT ceramics (*x* = 0–0.8), the values of activation energy are close to the double ionization activation energy of the oxygen vacancy at the lower frequencies of 1 kHz and 10 kHz, while the values of activation energy are close to the single ionization activation energy of the oxygen vacancy at the higher frequencies of 100 kHz and 1000 kHz. These results indicate that the conductivity mechanism is mainly related to the secondary ionization and single ionization of the oxygen vacancy at lower and higher frequencies, respectively. It also can be seen that the activation energy of BiT ceramics is very low, between 0.05 and 0.38 eV, indicating that its conductive mechanism is no longer oxygen vacancies, the conductive mechanism is probably impurity conductance (Zhang, S. J et al., 2005). Thus, the oxygen vacancies are the main carriers and the main cause of the conductivity behavior for (1-*x*) BBT—*x* BiT ceramics (*x* = 0–0.8), and the conductive mechanism of BiT ceramics is probably impurity conductance.

## 4 Conclusion

In this study, (1-*x*) BaBi_4_Ti_4_O_15_—*x* Bi_4_Ti_3_O_12_ ceramic samples with intergrowth bismuth layer structure were fabricated by a conventional solid-state reaction method. The results of XRD and TEM show that the BBT-BiT intergrowth ceramics formed the intergrowth structure of two kinds of perovskite layers along the *c*-axis, and there is no obvious second phase. The construction of this intergrowth structure leaded to a large reduction in the oxygen vacancy concentration in this samples through the XPS results, and the reduction in the oxygen vacancy concentration will inhibit grain growth as shown in SEM results. It is also proposed that the change of oxygen vacancy concentration may affect the relaxation activation energy, the deflection of ferroelectric domains and the piezoelectrical properties. Furthermore, the intergrowth structure of BLSFs affects the symmetry of ceramic *c*-axis direction, which has an impact on the electrical properties of the ceramics. Based on the above influence, the 0.8BBT-0.2BiT intergrowth ceramics show the best performance: *d*_33_ = 16 pC/N, *T*_c_ = 458°C, *σ*_ac_ = 5.6 × 10^–5^–6.8 × 10^–3^ S/m (*T* < 475°C, *f* = 10^2^–10^6^ Hz).

## Data Availability

The original contributions presented in the study are included in the article/supplementary material, further inquiries can be directed to the corresponding author.
